# Application of response surface methodology for obtaining fermented extract of *Sanguisorba officinalis* L. herb with high antioxidant activity, polyphenols, and lactic acid content: analysis of the content and skin permeation of selected phenolic acids

**DOI:** 10.1039/d5ra06662j

**Published:** 2025-11-17

**Authors:** Anna Muzykiewicz-Szymańska, Edyta Kucharska, Łukasz Kucharski, Robert Pełech, Anna Nowak

**Affiliations:** a Department of Cosmetic and Pharmaceutical Chemistry, Pomeranian Medical University in Szczecin 72 Powstancow Wlkp. Ave. 70-111 Szczecin Poland anna.muzykiewicz@pum.edu.pl; b Department of Chemical Organic Technology and Polymeric Materials, West Pomeranian University of Technology in Szczecin 10 Pulaski Str. 70-322 Szczecin Poland

## Abstract

Fermented plant extracts are becoming increasingly popular as active ingredients in cosmetics and pharmaceutical preparations applied topically to the skin. One of the most popular fermentation methods is fermentation using lactic acid bacteria. The aim of the research was to assess the impact and optimise the technological parameters of the fermentation process (using response surface methodology) of the *Sanguisorba officinalis* L. herb (*i.e.*, fermentation time, plant material content, molasses content, and inoculum content) in terms of antioxidant activity (DPPH and FRAP techniques), total polyphenol content, and lactic acid content. Using optimal parameters – fermentation time: 10 days, plant material content: 1.3 g L^−1^, molasses content: 16%, and inoculum content: 19% – a fermented extract was obtained with antioxidant activity of 8.1 ± 0.3 mmol Trolox per L (DPPH method), 44.1 ± 0.1 mmol FeSO_4_ per L (FRAP method), polyphenol content of 3.2 ± 0.1 g GA per L and lactic acid (LA) content of 25.2 ± 1.1 g L^−1^. The obtained fermented extract is characterized by high hydrophilicity (log *P* = −0.8). HPLC analysis showed the presence of phenolic acids in the fermented extract, among which the highest concentrations were observed for chlorogenic acid (347 ± 7 mg L^−1^), 4-hydroxybenzoic acid (231 ± 8 mg L^−1^), 2,5-dihydroxybenzoic acid (210 ± 10 mg L^−1^), and gallic acid (205 ± 6 mg L^−1^). Among the identified acids, gallic acid permeated porcine skin the fastest and achieved the highest concentration, ranging from 11.62 ± 1.05 µg cm^−2^ at the 5th hour of penetration to 35.8 ± 1.6 µg cm^−2^ after 24 hours. The presence of 3,4-dihydroxybenzoic acid, 2,5-dihydroxybenzoic acid, 4-hydroxybenzoic acid, and vanillic acid was also noted in the acceptor fluid after 24 hours of permeation. The obtained results confirm the validity of using the fermentation process to obtain extracts with a high content of compounds with antioxidant potential, including polyphenols and lactic acid, which have a multifaceted positive effect on the skin. Optimisation of the fermentation process of the *S. officinalis* herb, which has so far had very limited use in cosmetic and pharmaceutical preparations (unlike the root of this plant), may contribute to the increased interest in and use of this plant raw material in the cosmetic and pharmaceutical industries.

## Abstract 2

Optimised fermented extract of the herb *Sanguisorba officinalis* L. with high antioxidant activity, polyphenols, and lactic acid content.

## Graphical abstract

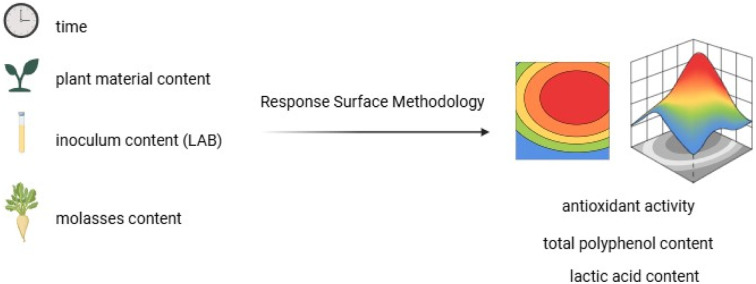

## Introduction

Over the years, we have observed an increase in interest in the use of natural raw materials in various industries, including the food, cosmetics, and pharmaceutical industries.^1^ Natural substances in preparations applied to the skin can have anti-ageing effects, brighten the skin, protect against UV radiation, or moisturise it. The literature frequently highlights the antioxidant, anti-inflammatory, and antimicrobial potential of raw materials of plant origin.^2^

One of the plants that may be used in preparations applied topically to the skin is *Sanguisorba officinalis* L. (Rosaceae family), also known in the literature as great burnet. The root of this plant is mainly used as a pharmaceutical raw material, which has a number of activities, including antioxidant, anti-cancer, anti-inflammatory, anti-lipid peroxidation, antibacterial, hepatoprotective, anti-diabetes, and anti-obesity effects.^3,4^ The root extract of this plant is also registered as a cosmetic ingredient (INCI Name: *Sanguisorba officinalis* root extract) with cleansing, refreshing, skin conditioning, and tonic functions (data from the COSMILE Europe database^5^). Although the root of this plant is mainly used as a pharmaceutical and cosmetic raw material, previous studies by Muzykiewicz-Szymańska *et al.* have shown that ethanol extracts from the herb of this plant are a valuable source of antioxidants, including polyphenolic compounds that have the ability to permeate through the epidermis and accumulate in the skin.^6^ Subsequent research by this team focused on optimising the ultrasound-assisted extraction process of *Sanguisorba* herba in terms of its high antioxidant activity (assessed by DPPH and FRAP techniques) and total polyphenol content. For optimisation, the authors used Response Surface Methodology (RSM).^7^ RSM is a frequently used method to optimise the extraction process of plant raw materials (both conventional and modern)^8–10^ as well as to obtain fermented extracts.^11–13^ Multifactorial statistical methods for modelling and optimising the extraction processes of bioactive compounds reduce the number of experiments performed, which in turn lowers the costs and time required for research. Moreover, the multi-factor approach provides a better overview of the interaction effects between parameters and their impact on a specific process outcome.^14^

In addition to classic plant extracts (obtained both by conventional methods, *e.g.* extraction in a Soxhlet apparatus or maceration, and modern ones, *e.g.* extraction assisted by ultrasound or microwaves), fermented extracts are increasingly used. Fermented plant extracts are the product of the fermentation process of plant material by appropriately selected strains of bacteria or fungi in precisely adjusted conditions. In the process of obtaining fermented plant extracts, the most commonly used strains are Lactic Acid Bacteria (LAB), *e.g.*, *Lactobacillus plantarum*, *L. rhamnosus*, *L. brevis*, *L. casei*, *L. gasseri*, *L. bulgaricus*, *L. reuteri*, and various types of *Bacillus* bacteria strains, *e.g.*, *B. amyloliquefaciens*, *B. methylotrophicus*, and *B. subtilis*. It is also possible to use fungal strains such as *Aspergillus oryzae*, *A. usamii*, *A. niger*, or *Saccharomyces cerevisiae*. It has been confirmed that the fermentation process can increase the compatibility, biological effectiveness and bioavailability of extracts while reducing their cytotoxicity. The fermentation process converts high-molecular-weight compounds into low-molecular-weight ones, which results in the release of particles from the substrate that are more accessible and susceptible to penetrating the stratum corneum, then through the remaining layers of the epidermis up to the dermis.^15–17^ Microorganisms involved in the fermentation process not only lead to the disintegration of the cell walls of plant materials but also, thanks to their hydrolytic activity, increase the concentration of certain active compounds, *i.e.*, flavonoids, anthocyanins, organic acids, proteins, ceramides, amino acids and enzymes.^11^ In the context of antioxidant potential, the ability of, *e.g.*, lactic acid bacteria to transform polyphenolic compounds seems to be particularly important. Lactobacillaceae possess a broad spectrum of enzymatic activity for the biotransformation of bioactive phenolic compounds. The conversion of phenolic compounds occurs with the participation of various types of decarboxylases, esterases, hydrolases and reductases. Plants are a particularly valuable source of flavonoids, which generally occur in the form of glycosides. In the fermentation process with lactic acid bacteria, glycosyl hydrolases target the sugar molecules attached to flavonoids, releasing the aglycons in the process.^18^*S. officinalis* leaves are also a source of phenolic glycosides, such as quercetin-glucoside, quercetin-galloyl-glucoside, and caffeic acid-glucoside. In addition, this plant material is a source of phenolic acids, such as neochlorogenic acid, chlorogenic acid, and *p*-coumaroylquinic acid.^19^ The conversion of phenolic glycosides into free polyphenols increases their bioavailability. Lactobacillaceae show enzymatic activity not only towards flavonoids but also towards phenolic acids (both hydroxybenzoic and hydroxycinnamic acids).^11^ The consequence of increasing the level of polyphenolic compounds during the fermentation process is an increase in the antioxidant activity of the obtained extracts. This feature is one of the advantages of using the fermentation process to obtain active ingredients in preparations applied topically to the skin.^20^ Preparations for topical application to the skin containing plant extracts with high antioxidant potential are particularly useful in the care of mature skin. They influence, among others, the improvement of overall skin elasticity and the reduction of melanin and erythema.^21^ The environmental benefits of fermented ingredients over traditional chemical ones include using renewable materials, simpler production processes, less waste, and biodegradability.^15,16^ In the presented research, the fermentation process was performed using *Lactobacillus* bacteria. Lactic acid fermentation is carried out at low temperature and mild conditions, thus reducing the risk of degradation of active ingredients. In addition, extracts obtained by fermentation with *Lactobacillus* strains contain metabolites that have a therapeutic and cosmetic effect on the skin.^22^ One of the products of the fermentation process with *Lactobacillus* strains is lactic acid, which, by lowering the pH of the process, contributes to the production of short-chain fatty acids and other organic acids. Lactic acid also helps maintain the physiological, slightly acidic pH of the skin, which protects against the development of pathological bacterial flora and supports the skin microbiome. It also has a gentle exfoliating and skin-brightening effect. Additionally, lactic acid is a natural preservative.^23^ Importantly, lactic acid is a component of the Natural Moisturising Factor, which is one of the elements ensuring proper skin hydration.^24^ The presented research was dictated by the limited number of studies on the *S. officinalis* herb, despite the content of valuable active compounds, especially those with antioxidant potential. To our knowledge, no studies have been published on fermented extracts of the *S. officinalis* herb, in particular focusing on the optimisation of the fermentation process parameters of this plant material. The aim of the research was to assess the impact and optimise the technological parameters of the fermentation process (*i.e.*, fermentation time (*t*), plant material content (PMC), molasses content (MC), and inoculum content (Inoc)) in terms on antioxidant activity (AA-DPPH and AA-FRAP), total polyphenol content (TPC), and lactic acid content (LAc). The content of selected phenolic acids in the fermented extract was assessed using HPLC. The dermocosmetic potential was confirmed by preliminary *ex vivo* studies, in which the ability of the identified phenolic acids to permeate porcine skin was analysed.

## Experimental

### Material and reagents

#### Plant material

Dried and cut *Sanguisorba officinalis* L. herb (EkoHerba, Hajnówka, Poland) was ground in a laboratory grinder and sieved through laboratory sieves (MULTISERW-Morek, Brzeźnica, Poland) with a diameter of 0.25 to 0.63 mm. A fraction of the raw material with a diameter below 0.25 mm was used for the fermentation process.

#### Fermentation process

OXOID (M.R.S. BROTH, Rogosa, Sharpe) supplied a LAB medium (CM0359, a non-selective medium for the abundant growth of lactic acid bacteria). Probiotics (Novara, Italy) provided the lactic acid bacteria strains *L. salivarius* LY_0652, *L. reuteri* MI_0168, *L. brevis* LY_1120, *L. rhamnosus* MI-0272, *L. plantarum* MI-0102, and *L. acidophilus* MI-0078. Lipase AY30 was obtained from Thermo Scientific (Białystok, Poland), whereas the BIO cane molasses (NatVita) were purchased from Mirków, Poland. Chempur (Piekary Śląskie, Poland) delivered potassium dihydrogen phosphate, ammonium sulphate, and calcium chloride. All reagents were of analytical grade.

#### Antioxidant activity analysis

DPPH· (2,2-diphenyl-1-picrylhydrazyl), Trolox (6-hydroxy-2,5,7,8-tetramethylchroman-2-carboxylic acid), Folin–Ciocalteu reagent, gallic acid, TPTZ (2,4,6-tris(2-pyridyl)-*s*-triazine) from Merck (Darmstadt, Germany). Hydrochloric acid, ethanol, methanol, 99.5% acetic acid, iron(iii) chloride, iron(ii) sulfate, sodium carbonate and sodium acetate anhydrous from Chempur, Piekary Śląskie (Poland). All reagents were of analytical grade.

#### HPLC analysis and the permeation study

Glacial acetic acid from Chempur, Piekary Śląskie (Poland). Methanol from JT Baker, Phillipsburg (USA). Gallic acid, 4-hydroxybenzoic acid, and PBS from Merck, Darmstadt (Germany). 3,4-dihydroxybenzoic acid, 3-hydroxybenzoic acid, 2,5-dihydroxybenzoic acid, ferulic acid, and vanillic acid from Sigma-Aldrich Chemie GmbH, Steinheim (Germany). Chlorogenic acid from Pol-Aura, Zawroty (Poland). All reagents were of analytical grade. The porcine skin, as a food waste, was purchased from a local butcher (Agrofirma Witkowo, Szczecin, Poland).

### Preparations of fermented extract of *S. officinalis* herb (FSE). Antioxidant activity (AA) and total polyphenol content (TPC) analysis

Fermented *Sanguisorba* extracts (FSEs) were obtained by the fermentation of *S. officinalis* herb. In our study, as a raw material to produce lactic acid, we used BIO cane molasses, which is relatively inexpensive and readily available and enables high fermentation efficiency towards the main product, *i.e.*, lactic acid, compared to other raw materials such as glucose or starch.^25^ We measured the total amount of 6-carbon sugars (Brix) in the cane molasses using a refractometer (KRUSS Optronic DR301-95, A. Kruss Optronic GmbH). Next, the fermentation was carried out using an inoculum consisting of a mixture of LAB strains: *L. salivarius* LY_0652, *L. reuteri* MI_0168, *L. brevis* LY_1120, *L. rhamnosus* MI-0272, *L. plantarum* MI-0102, and *L. acidophilus* MI-0078,^26^ which were prepared according to a previously used procedure.^11^ The fermentation process was carried out in 100 mL glass bioreactors, into which the raw materials were introduced in the following order: cane molasses (the amount of which was controlled depending on the process parameter under study while maintaining an initial sugars content of 5–20%), deionized water, herb of *S. officinalis* (the amount of which was controlled depending on the process parameter 0.1–2.0 g L^−1^), and mineral salts such as potassium dihydrogen phosphate, ammonium sulphate, and calcium chloride. The contents of the bioreactor were mixed for 2 minutes, until the cane molasses and mineral salts were fully dissolved. Then the LAB inoculum was added (the amount of which was controlled depending on the process parameter under study while maintaining a content of 5–25%). The fermentation process was carried out at a temperature of 37 ± 0.5 °C, and its duration was monitored. The process was completed after 14 days.^27^ The highest concentration of lactic acid was recorded after 14 days of fermentation. Extending the process did not result in a significant increase in its content, which indicates that a plateau in LA production has been reached. Two independent experiments carried out the fermentation process. After finishing the process, lipase was added to hydrolyse the bacterial cell wall, and the resulting FSEs were filtered with a glass funnel, spun in a centrifuge for 9 minutes, and then filtered again using sterile syringe filters with a pore size of 0.45 µm, which are used to sterilise liquid solutions. In this way, the fermented plant extract was free of microorganisms.^28^ FSEs were stored in a freezer at −10 °C until analysis.

The antioxidant activity of the FSEs was assessed by the DPPH (AA-DPPH) and FRAP (AA-FRAP) methods, while the total polyphenol content (TPC) was assessed by the Folin–Ciocalteu technique. The Hitachi U-5100 UV-Vis spectrophotometer (Tokyo, Japan) performed the analyses in accordance with the methodology described previously by Muzykiewicz-Szymańska *et al.*^7^ AA-DPPH results were expressed as Trolox equivalents [mmol Trolox per L], AA-FRAP in mmol FeSO_4_ per L and TPC in gallic acid equivalents [g GA per L]. All results are presented as the arithmetic mean of three independent samples ± SD (standard deviation).

### Lactic acid content (LAc) – GC-MS analysis

The gas chromatography-mass spectrometry (GC-MS) analysis was carried out with a Shimadzu GCMS-QP2020 NX with a Shimadzu SH-I-5MS column (30 m × 0.25 mm × 0.25 µm) similarly to the prior research.^29^ All samples were analysed three times. The concentration of lactic acid (LAc) in FSE was calculated in the same way as in the previous study,^29^ based on the obtained calibration curve using octane as an internal standard. Results are presented as the mean ± SD.

### Response surface method optimisation

Mathematical optimisation of the fermentation process of *S. officinalis* herb using cane molasses (whose total content of 6-carbon sugars was 69%) in the presence of lactic acid bacteria strains was carried out according to the described procedure using a fractional plan.^11^ To get the most adequate mathematical description of the course of the fermentation process of *S. officinalis*, four technological parameters are used: fermentation time, inoculum content, the content of molasses calculated on the basis of the initial sugar concentration, and plant material content. Actual input values were used for the calculations.

Based on the experimental results, a significance analysis was performed, and the regression functions of the response surface describing the fermentation process of *S. officinalis* herb were determined, while the optimal process parameters were determined based on the determined regression functions. The ranges of variation of the input values were as follows: *X*_1_ (fermentation time, *t*) 2–14 days, *X*_2_ (inoculum content, Inoc) 5–25%, *X*_3_ (the content of molasses calculated on the basis of the initial sugar concentration, MC) 5–20%, and *X*_4_ (plant material content, PMC) 0.1–2.0 g L^−1^. The main functions describing the fermentation process were antioxidant activity (AA-DPPH and AA-FRAP) assessed by the DPPH and FRAP methods; total phenolic compounds (TPC) assessed by the Folin–Ciocalteu method; and lactic acid content (LAc) assessed by the GC-MS method.

The experimental design and results of each experiment are presented in Table S1. The independent variables were coded according to the following eqn (1), which allows for their comparability and statistical analysis within the regression model:1
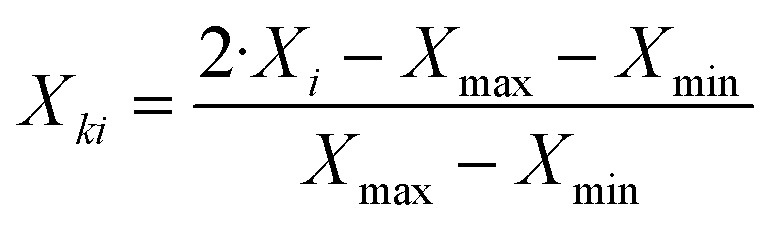


Multiple regression analysis was used to develop a model describing the influence of independent variables on the fermentation process responses. This method allows for determining the significance of individual factors and their interactions, as well as determining regression equations describing the response surfaces.^30^ Statistical analyses were performed on data expressed in real form, which enabled direct interpretation of the influence of individual process variables on the model responses.

The influence of normalised independent factors (*X*_*i*_, *X*_*j*_) of the *S. officinalis* herb fermentation process on the value of the response function (*Y*_*i*_) was presented using a second-degree algebraic polynomial (2):2
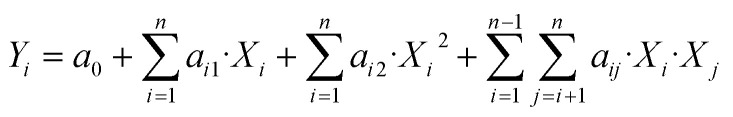
where: *X*_*i*_ – fermentation time, *t* (1); inoculum content, Inoc (2); the content of cane molasses calculated based on the initial sugar concentration, MC (3); and plant material content, PMC (4); *a*_*i*_ – regression coefficients; *Y*_*i*_ – the relevant dependent variable: the antioxidant activity, AA-FRAP (1); the antioxidant activity, AA-DPPH (3); total polyphenol content, TPC (2); and lactic acid content, LAc (4).

The coefficients of the regression equations for the normalised input quantities were determined by the least square's method using matrix calculus (Table S2). Fig. S1A–D shows the scatter distribution between the approximated and observed values for antioxidant activity by FRAP (AA-FRAP) (Fig. S1A), AA-DPPH (Fig. S1B), TPC (Fig. S1C), and LAc (Fig. S1D). Fig. S1A shows a plot of residuals (*i.e.*, the differences between the values predicted by the regression model and the actual experimental values) for antioxidant activity determined by FRAP. The even distribution of points around the lines indicates good model fit. The high correlation coefficient *R*^2^ = 0.977 and the adjusted Adj *R*^2^ = 0.972 for AA-FRAP confirm that the model describes the data variability very well (Table S2). A similar relationship can be observed for AA-DPPH (*R*^2^ = 0.948 and Adj *R*^2^ = 0.937, Fig. S1B), TPC (*R*^2^ = 0.973 and Adj *R*^2^ = 0.967, Fig. S1C), as well as lactic acid content (*R*^2^ = 0.908 and Adj *R*^2^ = 0.888, Fig. S1D). Table 1 presents the extreme values of the independent variables (fermentation time, inoculum content, the content of molasses, and plant material content) and the corresponding response values: AA-FRAP, TPC, AA-DPPH, and LAc determined for the fermentation process. It was found that the function describing the AA-FRAP value reaches an extreme outside the range of variability of the input variables, *i.e.*, it does not reach a local maximum or minimum within the analysed range of variable values. Within this range of variability, the FRAP value is an increasing function of the studied parameters. However, for AA-DPPH, TPC, and LAc, the functions reach extremes within the analysed range, allowing us to determine optimal fermentation conditions to maximize the response values.

**Table 1:** Extreme values of independent variables and their corresponding response values: AA-FRAP, TPC, AA-DPPH and LAc^a^

	AA-FRAP	TPC	AA-DPPH	LAc
*t*	−31	11	10	9.9
Inoc	174	20	19	19
MC	−50	17	16	16
PMC	−4.3	1.4	1.3	1.3
Extreme response	−55	33	7.5	25

a *t* – Fermentation time; Inoc – inoculum content; MC – molasses content; PMC – plant material content.

#### Lipophilicity assessment

The lipophilicity assessment of the optimised FSE (for the sum of active compounds with absorbances in the wavelength range of 190–450 nm) was performed according to the previously developed methodology and based on the following eqn (3):^7^3
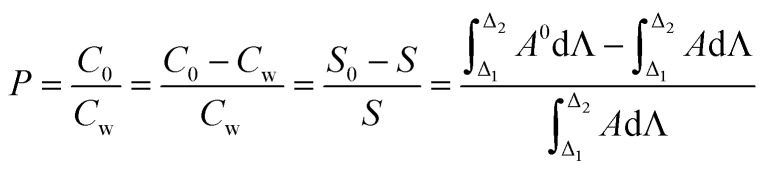
where: *C* – concentration of total compounds in the *n*-octanol layer and in the water layer; *S* – area occupied by the compound in the UV-Vis spectrum; *A* – absorbance; 0 – concentration of total compounds in the *n*-octanol layer and in the aqueous layer concentration of total compounds in the *n*-octanol layer and in the aqueous layer/initial area occupied by the compound in the UV-Vis spectrum/initial absorbance; *Λ* – wavelength.

Based on the mass balance and the assumption that the Lambert–Beer law is fulfilled in the tested wavelength range (190–450 nm), the ratio of the concentrations of active compounds contained in the optimised FSE was determined using a Thermo Scientific GENESYS 50 spectrophotometer (Thermo Fisher Scientific, Norristown, USA).^31^

### HPLC analysis and *ex vivo* skin permeation study

Analysis of selected phenolic acids in FSE and acceptor fluid collected during the permeation study was performed according to a slightly modified method described by Jakubczyk *et al.*^32^ using a Knauer (Berlin, Germany) HPLC system coupled with a WellChrom UV K-2600 detector. The 150 × 4 mm (C18) column packed with Hypersil GOLD with a 3 µm particle size was used for analysis, and detection was performed at *λ* = 278 nm. Compounds were identified based on retention times by comparison with a standard under the same conditions. HPLC analysis was performed in gradient mode using 1% aqueous acetic acid (A) and methanol (B) as the mobile phase. The gradient program was as follows: 90% A/10% B (0–6 min), 84% A/16% B (7–25 min), 72% A/28% B (26–37 min), 65% A/35% B (38–47 min), 50% A/50% B (48–64 min), and then back to 90% A/10% B (65–70 min). The flow rate was 0.8 mL min^−1^, the injection volume was 20 µl, and the temperature was maintained at 25 °C. The presented results are the arithmetic mean (mean ± SD) of three measurements.

The permeation of selected phenolic acids was studied according to a slightly modified methodology described in the publication by Ziemlewska *et al.*^33^ using Franz diffusion cells (Phoenix DB-6, ABL&E-JASCO, Vienna, Austria). This apparatus is equipped with acceptor cells with a capacity of 10 cm^3^ filled with PBS solution at pH 7.4, while donor cells have an area of 1 cm^2^, to which 1 cm^3^ of FSE was applied. During the analysis, a constant temperature of 37.0 ± 0.5 °C and a stirring speed of 350 rpm were maintained in the diffusion units. Porcine skin, which has similar properties and structure to human skin, was used for the study.^34^ Ziemlewska *et al.*^33^ published a detailed description of the method of skin preparation and integrity testing. The permeation study was conducted for 24 hours. At specific time points (1, 2, 3, 5, 8, and 24 hours), 0.5 cm^3^ samples of the acceptor fluid were collected and replaced with a fresh portion of PBS. The collected samples were analysed for phenolic acid content using HPLC. The presented results represent the arithmetic mean (mean ± SD) of three measurements.

### Statistical analysis

The fractional plan was used to optimise the fermentation process of *S. officinalis* L. herb using cane molasses and lactic acid bacteria strains. We used the program Statistica 13.3 PL software 7 (StatSoft, Kraków, Poland) to create the experimental plan, statistical computations, and contour drawings. A one-way analysis of variance (ANOVA) was used to statistically analyse the optimisation process. We used an ANOVA test to check the adequacy of the tested function. Results are displayed as the mean ± SD.

## Results and discussion

The main objective of this study was to optimise selected parameters of the great burnet herb fermentation process in terms of antioxidant activity determined by DPPH and FRAP, as well as total polyphenolic and lactic acid content. Key parameters, such as the duration of the fermentation process and the concentration of its substrates—plant material, sugar (molasses), and inoculum—were optimised. For the fermentation process to be effective and efficient, it is essential to select appropriate substrate concentrations. Both too low and too high nutrient and substrate concentrations can reduce the efficiency and productivity of LA, which in turn negatively impacts the extract's antioxidant potential. Too low substrate concentrations may contribute to premature depletion of substrates in the fermentation broth, while too high concentrations may lead to longer lag phases, osmotic stress, cell lysis, and decreased microbial activity. The inoculum size (the microbial population introduced into the fermentation medium) typically ranges from 5 to 20%. Larger inoculum sizes used in the fermentation process result in faster and higher concentrations of LA production. This is due to the fact that a larger inoculum size leads to a lower initial pH, which facilitates and accelerates the accumulation of LA. Therefore, one key issue in optimising the fermentation process is analysing the interaction between substrate and inoculum concentrations.^35,36^ The duration of the fermentation process is another very important factor influencing the content of compounds with antioxidant potential in the final extract, as well as lactic acid. Fermentation is a dynamic process that involves the growth and metabolism of microorganisms, as well as the degradation of various substances. Both too short and too long a time will negatively impact the properties of the resulting extract.^11,37^

**Fig. 1:**
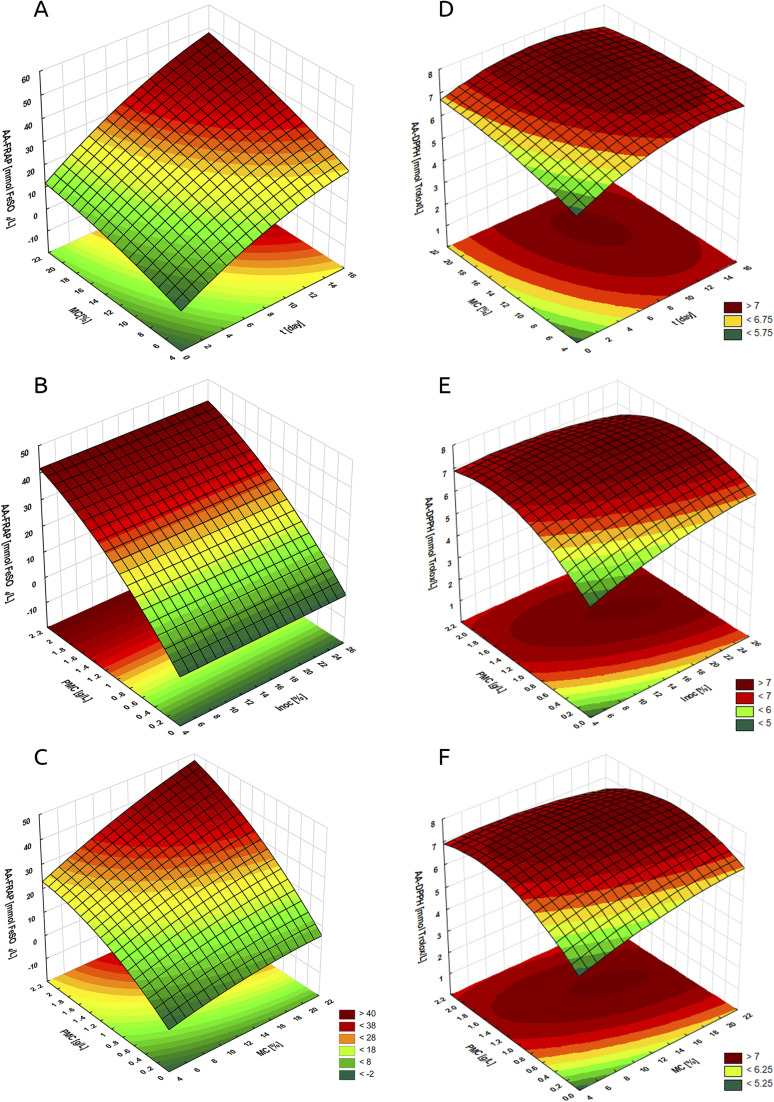
Changes in antioxidant activity determined by the FRAP method (AA-FRAP, A–C) and by the DPPH method (AA-DPPH, D–F). Relationship: (A and D): molasses content (MC) and fermentation time (*t*) – constant parameters of the process: inoculum content 19% and plant material content 1.3 g L^−1^; (B and E): plant material content (PMC) and inoculum content (Inoc) – constant parameters of the process: fermentation time 10 days and molasses content 16%; (C and F): plant material content (PMC) and molasses content (MC) – constant parameters of the process: fermentation time 10 days and inoculum content 19%.

**Fig. 2:**
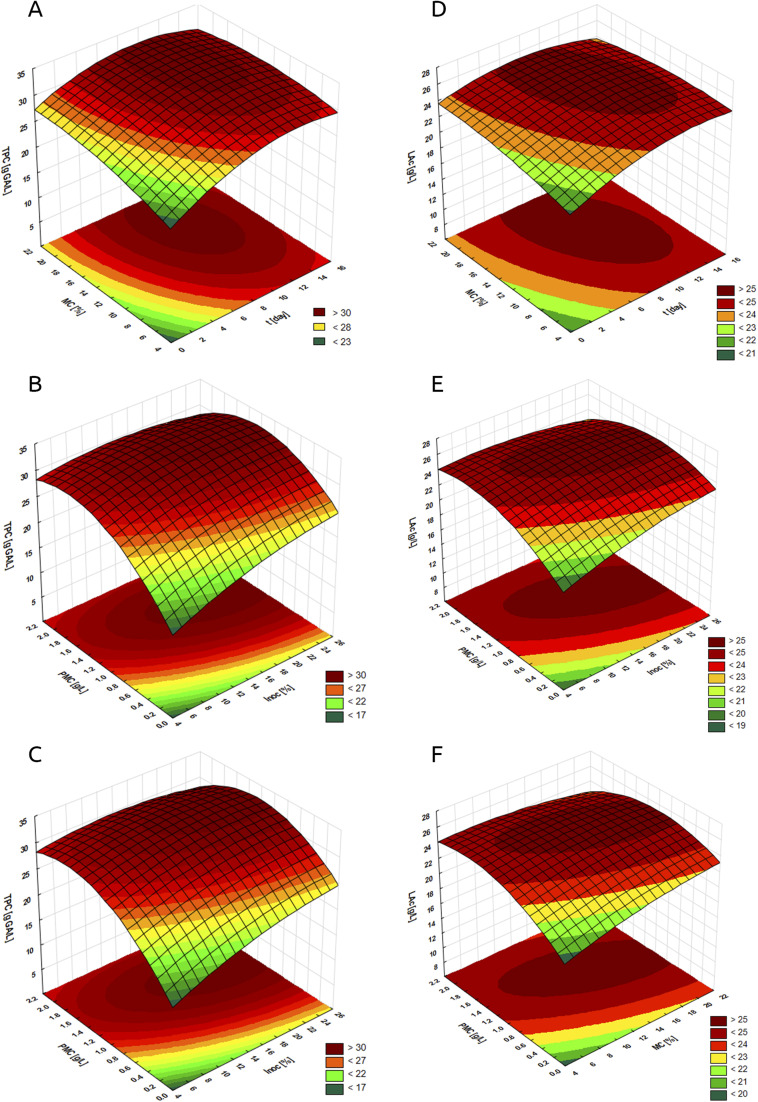
Changes in total polyphenol content (TPC, A–C) and lactic acid content (LAc, D–F). Relationship: (A and D): molasses content (MC) and fermentation time (*t*) – constant parameters of the process: inoculum content 19% and plant material content 1.3 g L^−1^; (B and E): plant material content (PMC) and inoculum content (Inoc) – constant parameters of the process: fermentation time 10 days and molasses content 16%; (C and F): plant material content (PMC) and molasses content (MC) – constant parameters of the process: fermentation time 10 days and inoculum content 19%.

Table 2 presents the optimal parameters of the *S. officinalis* herb fermentation process using LAB, along with the corresponding values of the main process functions. The reliability of the RSM for the fermented plant extract obtained from *S. officinalis* herb was confirmed experimentally (Table 3). Fig. 1 and 2 present the dependencies of the optimised parameters (*t*, PMC, MC, Inoc) on the antioxidant activity determined by the FRAP method (Fig. 1A–C), DPPH (Fig. 1D–F), total polyphenol content (Fig. 2A–C), and lactic acid content (Fig. 2D–F).

Optimal parameters for the fermentation process of *S. officinalis* herb in the presence of LAB and cane molasses and the corresponding values of the main functions of the process

**Table:** 

Optimal parameters of the fermentation process	Unit	Value
Fermentation time	day	10
Inoculum content	%	19
Molasses content	%	16
Plant material content	g L^−1^	1.3

**Table:** 

Functions of the fermentation process	Unit	Value
TPC	g GA per L	3.3
AA-FRAP	mmol FeSO_4_ per L	27.5
AA-DPPH	mmol Trolox per L	7.54
LAc	g L^−1^	25.4

**Table 3:** The values for the main functions of the *S. officinalis* fermentation process according to the RSM analysis

Functions of the fermentation process^a^	Unit	Value
TPC	g GA per L	3.2 ± 0.1
AA-FRAP	mmol FeSO_4_ per L	44.1 ± 0.1
AA-DPPH	mmol Trolox per L	8.1 ± 0.3
LAc	g L^−1^	25.2 ± 1.1

a Constant parameters of the process: fermentation time 10 days; inoculum content 19%; molasses content 16%; raw material content 1.3 g L^−1^.

The optimal parameters of the fermentation process in terms of antioxidant activity (AA-DPPH and AA-FRAP), polyphenol content and lactic acid determined as the average of the input values corresponding to the extremes of the tested parameters, were: *t* = 10 days, Inoc = 19%, MC = 16% and PMC = 1.3 g L^−1^ (Table 2). The reliability of the response surface methodology for the fermented plant extract obtained from *S. officinalis* herb was confirmed experimentally, with the following values of the main process functions obtained: AA-DPPH = 8.1 ± 0.3 mmol Trolox per L; TPC = 3.2 ± 0.1 g GA per L, AA-FRAP = 44.1 ± 0.1 mmol FeSO_4_ per L, and LAc = 25.2 ± 1.1 g L^−1^ (Table 3). Kucharska *et al.*^11^ optimised the time, content of plant material and initial sugar content (beet molasses) in the fermentation process of clove buds (*Syzygium aromaticum* L.) in the presence of LAB. They used antioxidant activity (determined by the DPPH method), total polyphenol content and lactic acid as functions of the fermentation process. In the cited study, the optimal fermentation process time was 9 days, which is a similar value to that in our study, while the optimal PMC and MC differed significantly. The optimal content of clove buds was 6.4% (64 g L^−1^), while molasses and inoculum were 3.2%. Using optimal fermentation parameters, an extract was obtained whose antioxidant activity, determined by the DPPH method, was 33.9 mmol Trolox per L, polyphenol content 11.6 mmol GA per L (1.97 g GA per L), and lactic acid content 96%. In our study on the optimisation of the fermentation process of the great burnet herb during 10 days, using 1.3 g of herb per L, 19% inoculum content, and 16% molasses, we obtained an extract with lower AA-DPPH (8.1 mmol Trolox per L) but with a higher polyphenol content (3.2 g GA per L). In our extract the lactic acid content was at a lower level (25%). However, it is worth noting that in the study by Kucharska *et al.*^11^ the solid : solvent ratio was approximately 1 : 15, while in our study it was approximately 1 : 770, *i.e.*, from almost 50 times less plant material a fermented extract with a higher content of polyphenols, which are the most important group of plant-derived antioxidants, was obtained. It is highly probable that one of the reasons for the high extraction efficiency of compounds with antioxidant potential, including polyphenols, was the grinding of the plant material prior to the fermentation process. A similar relationship was observed in studies conducted by Muzykiewicz-Szymańska *et al.*^7^ In this study, the process of ultrasound-assisted extraction of the great burnet herb was optimised in terms of antioxidant activity (AA-DPPH, AA-FRAP) and TPC. The extraction parameters optimised in this study were the following: plant material content: 7.5 g/100 mL (75 g L^−1^), ethanol concentration used as a solvent: 47% v/v, and extraction time—11 minutes. The extract prepared using optimal parameters showed an activity of 12.7 mmol Trolox per L (DPPH) and 20.8 mmol FeSO_4_ per L (FRAP) as well as a TPC of 2.4 g GA per L. It should be noted that in the fermentation process of the great burnet herb, a 25% higher total polyphenol content was obtained from more than 55 times less content of plant raw material. The antioxidant activity of the fermented extract, assessed by the FRAP method, was twice as high as that of the optimised ethanol extract. This confirms the postulates of many authors that the fermentation process is an effective method for obtaining plant extracts with high antioxidant activity, including polyphenol content.^38–42^ It should also be noted that the FSE obtained by us is distinguished by a relatively high antioxidant activity and polyphenol content compared to extracts fermented from other plants, prepared under similar conditions to those used in the presented study. In another study by Kucharska *et al.*,^28^ the antioxidant activity of fermented *Silybum marianum* extract (fermentation time 21 days, PMC approx. 15 g L^−1^, Inoc approx. 30 mL L^−1^) was examined and compared with non-fermented extract. Antioxidant activity (determined by DPPH, ABTS, and FRAP methods) as well as total polyphenol content were higher in the fermented extract. The antioxidant activity of the fermented and non-fermented extract assessed by the DPPH method was 1.19 and 0.91 mmol Trolox per L; by the ABTS method, 0.20 and 0.10 mmol Trolox per L; and by the FRAP method, 1.34 and 0.52 mmol FeSO_4_ per L, respectively. TPC in the fermented extract was approximately 1/3 higher than in the unfermented extract (0.92 and 0.61 mmol GA per L, respectively, *i.e.*, 0.16 and 0.10 g GA per L). The antioxidant activity of the FSE prepared by us was more than 6 times (DPPH method) and more than 30 times (FRAP method) higher than that of the fermented *S. marianum* extract, and the polyphenol content was almost 20 times higher. FSE also had approx. 5 times higher lactic acid content. In subsequent studies, Kucharska *et al.*^29^ subjected pomace, extract, oil, and seeds of milk thistle to the fermentation process (fermentation time 14 days, PMC approx. 60 g L^−1^, inoculum content approx. 28 mL L^−1^, and molasses content approx. 50 g L^−1^). The antioxidant activity of the bioferments prepared in this study ranged from 2.41 to 3.53 mmol Trolox per L (DPPH method) and 10.77 to 16.68 mmol FeSO_4_ per L (FRAP method). The polyphenol content ranged from 2.31 to 2.59 g GA per L. This study once again confirms that the fermentation process of the great burnet herb is very efficient (optimal PMC = 1.3 g L^−1^), especially in terms of TPC.

By analysing Fig. 1A, D, 2A and 2D it can be seen that the interaction between molasses content and fermentation time had an effect on the antioxidant activity assessed by FRAP (Fig. 1A), DPPH (Fig. 1D), TPC (Fig. 2A), and lactic acid content (Fig. 2D) in the tested FSEs. The response surface analysis shows that when fermenting with mixed LAB strains and using the highest molasses content (*i.e.*, 21–22%) at the longest fermentation time (15–16 days), the highest AA-FRAP is observed, exceeding 40 mmol FeSO_4_ per L (Fig. 1A). This suggests that optimal fermentation conditions for maximising AA-FRAP activity occur at high substrate (sugar) content and extended fermentation time, which may favour the intensive production of phenolic compounds with reducing potential.^43^ Lower activities assessed by the FRAP technique at short fermentation times and low molasses content may be related to the insufficient metabolic activity of LAB under these conditions.^44^ A similar relationship was observed for AA-DPPH (Fig. 1D). When using a molasses content in the range of 12–20% (for a fermentation time of 8–12 days), the highest activity assessed by the DPPH method was observed (above 7 mmol Trolox per L). This suggests that optimal fermentation conditions do not occur at extreme values of any of these parameters (MC and *t*), but at their moderate levels. When the process was carried out with the initial sugar content below 12% (regardless of the fermentation time), a decrease in AA-DPPH was observed (down to a level below 5.75 mmol Trolox per L at MC = 4%), which may be related to the limited production of metabolites with antioxidant properties.^38^ However, too high a concentration of sugars in the fermentation solution (*i.e.*, above 20%) also slightly reduced AA-DPPH to a level below 6.75 mmol Trolox per L (MC = 20–22%, *t* = 1 day) due to the osmotic effect inhibiting the growth of microorganisms, limiting the metabolic activity and biosynthesis of phenolic compounds.^45^ The highest TPC (above 3 g GA per L) was observed when using molasses content in the range of 10–22% for a fermentation time of 8–14 days, which indicates optimal fermentation conditions at moderate values of both parameters (Fig. 2A). Fermentation at too low a sugar content (below 10%), especially at a short fermentation time (0–6 days), led to a significant decrease in TPC (even below 2.3 g GA per L at MC = 4–5%), which may be related to the limited presence of enzymes producing phenolic compounds as metabolites.^46^ Molasses content and fermentation time also influenced the lactic acid content in FSEs (Fig. 2D). At molasses contents ranging from 6 to 22% (for fermentation times of 6 to 14 days), the highest LA concentration (above 25 g L^−1^) was observed. This indicates optimal fermentation conditions within moderate ranges of both parameters. Too low a sugar content (<6%) led to limited LA production, regardless of fermentation time. LA values in this case ranged from <21 g L^−1^ (at MC = 4% and *t* = 0.5 days) to 25 g L^−1^ (at MC = 6% and *t* = 16 days), which may be related to insufficient substrate content for lactic acid bacteria.^27,47^

The next analysed relationships were the content of plant material (PMC) and inoculum (Inoc) (Fig. 1B, E, 2B and 2E). AA-FRAP was strongly dependent on PMC but practically independent of inoculum content (Fig. 1B). The response surface analysis shows that when the process was conducted at a maximum PMC level of 2.1–2.2 g (in the entire tested range of inoculum content), the highest values of the tested process function are observed (above 40 mmol FeSO_4_ per L). Thus, the inoculum concentration did not significantly affect the ability of FSEs to reduce Fe^3+^ ions to Fe^2+^. For AA-DPPH, the highest activities (exceeding 7 mmol Trolox per L) were observed with PMC in the range of 1.2–1.6 g L^−1^ and Inoc 14–22% (Fig. 1E). This means that optimal fermentation conditions for maximising AA-DPPH occur with moderately high plant material and inoculum contents. Too low a content of any of these components led to a decrease in AA-DPPH (even to a level below 5 mmol Trolox per L). The great burnet herb is characterised by a rich phytochemical composition, including the presence of flavonoids and their glycoside forms, which have antioxidant properties.^19^ During fermentation, in the presence of LAB strains, these compounds undergo enzymatic transformations into more bioavailable low-molecular-weight products, such as phenolic acids.^40^ These acids, due to the presence of hydroxyl groups in the benzene ring and conjugated double bonds, have the ability to donate electrons, stabilising free radicals and reactive oxygen species (ROS), which translates into their strong antioxidant properties.^48^ Low PMC limits the number of macromolecular compounds available for transformation, resulting in lower antioxidant activity. In turn, insufficient inoculum content may lead to too few active LAB cells, which limits the efficiency of enzymatic conversion of flavonoids and their glycosides into phenolic acids.^49^ The highest TPC (>3 g GA per L) was obtained with PMC in the range of 1.2–1.8 g L^−1^ and an inoculum of 13–26%, suggesting that moderately high levels of both parameters are conducive to maximising this function (Fig. 2B). The plant material contains polyphenols in the form of complex glycosides with weaker (compared to phenolic acids) bioavailability.^50^ During fermentation with LAB, enzymatic transformations of these compounds occur (mainly through the action of β-glucosidases, esterases, and phenolic decarboxylases), which leads to the release of free phenolic acids, such as gallic, ferulic, *p*-coumaric, or caffeic acid.^51^ Their presence in the fermented extract increases TPC and influences the antioxidant activity of fermented extracts.^11^ As already mentioned, too low a content of plant material in the fermentation mixture limits the amount of available phenolic compounds that can be released or transformed, while too low a content of inoculum does not provide sufficient enzymatic activity of LAB, which may also limit the efficiency of phenolic transformations.^52^ The highest LA contents (>25 g L^−1^) were observed at PMC content of 0.7–1.4 g L^−1^ and inoculum content of 12–26% (Fig. 2E). A high inoculum content in the fermentation process promotes a lower initial pH, which facilitates and accelerates the accumulation of lactic acid and, as a result, leads to higher yields and productivity.^35^

The study also analysed the antioxidant activity, total polyphenol, and lactic acid content in the interaction between plant material and molasses content (Fig. 1C, F, 2C and 2F). The analysed interactions affected all tested functions. For AA-FRAP, we found activities over 40 mmol FeSO_4_ per L when both parameters were at their highest, specifically with a PMC of 2.2 g L^−1^ and an MC of 22% (Fig. 1C). In the case of AA-DPPH, the highest activities (above 7 mmol Trolox per L) were observed at slightly lower PMC of 1.0–1.8 g L^−1^ and MC of 14–20% (Fig. 1F). The highest TPC values (>3 g GA per L) were observed at PMC of 0.9–1.9 g L^−1^ and MC of 12–22% (Fig. 2C). The highest LAc (>25 g L^−1^) were observed after using *S. officinalis* herb at 0.7–1.4 g L^−1^ and molasses at 12–26% (Fig. 2F). The obtained results indicate the importance of the interaction between the concentration of molasses and the content of plant material, which determine the final level of LA, polyphenols, and antioxidant activity. Molasses is a by-product of the confectionery industry, which is a viscous, dark brown liquid that contains nutrients such as sugars, minerals, vitamins, proteins, and biologically active substances, including phospholipids, oligosaccharides, and phenols. Due to high sugar content (sucrose, fructose, and glucose), molasses supports the growth of fermenting bacteria, including LAB, which in turn intensifies the production of lactic acid. By lowering the pH, LA stabilises the phenolic compounds released by LAB and their enzymatic activity. The higher polyphenol content, in turn, increases the antioxidant activity of fermented extracts.^38,53,54^ Ran *et al.*^55^ evaluated the effect of fermentation temperature and time, the amount of lactic acid bacteria inoculated, and the amount of added sugar on the antioxidant activity and total polyphenol content of honeysuckle beverage (*Lonicera japonica* Thunb). They performed the analysis using a single-factor test and an orthogonal test. The optimal parameters in their process were temperature 37 °C, time 24 h, amount of inoculated *Lactiplantibacillus plantarum* and *Lactobacillus acidophilus* (1 : 1) 3%, and white granulated sugar 8%. In the cited study, fermented beverages showed the highest capacity to scavenge the DPPH radical (at the level of 95%) in the 24th hour of the process. Shorter and longer process durations (>40 h) resulted in a decrease in the tested activity to approximately 85%. The polyphenol content was also highest after 24 h of fermentation (7.53 mg GAE per g) and decreased slightly with increasing time. It should also be noted that the researchers observed higher antioxidant activity and polyphenol content when they used a mixture (1 : 1) of *L. plantarum* and *L. acidophilus* in the process than when they used single strains. When using bacterial monocultures, antioxidant activity decreased by 0.23–2.7% and TPC by 0.3–0.57 mg GAE per g. Similarly to our studies, Ran *et al.*^55^ demonstrated a significant effect of the above-mentioned fermentation process parameters on antioxidant activity and polyphenol content in the extracts, which confirms the validity of their optimisation. Lin *et al.*^56^ optimised the ginseng fermentation process in terms of antioxidant activity to use the bioferment as an active ingredient in functional cosmetics. The optimal parameters for the process using the *L. plantarum* 1.140 strain were a time of 35.5 hours and an inoculum size of 2.45%. It should be noted, however, that in the cited study, both optimised parameters—time and inoculum size—had no significant effect on the antioxidant activity assessed by the DPPH technique. A significant effect of time and inoculum content was observed when assessing antioxidant activity using the superoxide anion scavenging activity technique. The ability to scavenge the O_2_˙^−^ radical decreased both at too low an inoculation concentration and at too short a fermentation time. The highest activity assessed by this method was found after approx. 30 hours of the process using an inoculum of approx. 2.5%. Moreover, the fermentation process conducted by Lin *et al.*^56^ did not increase the content of polyphenols; on the contrary, the researchers noted a decrease in the content of these compounds. The decrease in polyphenol content during the fermentation process is a much rarer phenomenon than its increase. In exceptional situations, a decrease in the content of these compounds is observed, which can generally be explained by enzymatic or non-enzymatic oxidation, diffusion of soluble phenolic compounds into fermentation sweating (exudates), or condensation reaction with protein. In most cases, however, an increase in polyphenol content is observed, which is associated with the generation of metabolites and liberation of phenolics from the matrix that compensates for the degradation of parent phenolic compounds during the fermentation process.^57^ Lee *et al.*^39^ investigated the effect of various parameters, including inoculum concentration and fermentation time, on the yield and antioxidant activity of extracts from *Ulmus davidiana* var. *japonica* root bark. In this study, both the inoculation level and the process duration influenced the tested extract properties and process efficiency. Too short and too long fermentation times, as well as too low and too high inoculum content, resulted in a decrease in process efficiency and a reduction in enzymatic activity. The fermentation process also resulted in an increase in the antioxidant activity of fermented extracts compared to non-fermented ones, as well as a higher content of selected polyphenols, in particular catechins and epicatechins. Liu *et al.*^58^ optimised and analysed the influence of selected fermentation process parameters (including time and inoculum content) on the antioxidant activity and flavonoid content of dandelion (*Taraxacum officinale*) extracts. Fermentation times that were too short (<36 h) and too long (>48 h) resulted in a decrease in flavonoid content. Fluctuations in the concentration of this group of polyphenols were not significant with changes in inoculum concentration, so the researchers adopted an inoculum concentration of 10% as the centre point for the response surface optimisation test. It should be noted that the content of plant material is a much less frequently optimised and analysed parameter of the fermentation process, even though, as shown in our study, it significantly influences the antioxidant activity, polyphenols, and lactic acid content of the obtained extracts. Liu *et al.*^59^ optimised the fermentation process of pomegranate peel and *Schisandra chinensis* for ellagic acid content. The optimal fermentation parameters in their study were as follows: temperature 37 °C, time 33 h, solid : solvent ratio 1 : 20, and glucose concentration 3 g/100 mL. The authors observed a decrease in the ellagic acid content with both shortening and extending the fermentation time, in particular up to 60 h. A similar relationship of the decrease in the ellagic acid content was observed with too low a glucose concentration (<3 g/100 mL) and too high, in particular 12 g/100 mL. In the case of the solid : solvent ratio, lower ellagic acid content was observed when the plant material concentration was too low. Increasing this ratio above the optimal level (1 : 20) resulted in a subtle decrease in ellagic acid content. We made similar observations in our study, where too low a PMC resulted in significant declines in the tested properties, while using higher concentrations of plant material above the optimal value did not result in such a significant decline in activity.

The results of the measurement of lipophilicity showed that the value of the log *P* partition coefficient for the optimised FSE was −0.8, which proves the high hydrophilicity of the fermented extract (Fig. S2). The hydrophilicity of the studied fermented extract is related to the presence of numerous hydroxyl and carboxyl groups, which are characteristic of phenolic compounds and LA – the main products of LAB fermentation. The presence of these functional groups increases the molecules' ability to form hydrogen bonds with water, which translates into their good solubility in an aqueous environment.^60^ The optimised extract from *S. officinalis* herb in the study published by Muzykiewicz-Szymańska *et al.*^7^ was also characterised by hydrophilicity (log *P* −0.307). In these studies, the authors indicated that the hydrophilic nature of the ethanol extract of the great burnet herb may be due to the presence of phenolic acids (*i.e.*, gallic acid, 3,4-dihydroxybenzoic acid, 2,5-dihydroxybenzoic acid, vanillic acid, 2,3-dihydroxybenzoic acid, caffeic acid, and chlorogenic acid).

To initially confirm the dermocosmetic potential of the optimised FSE, a phenolic acid profile analysis was performed using HPLC (Table 4), and a preliminary *ex vivo* skin permeation study was performed to assess the ability of the identified phenolic acids to permeate porcine skin (Table 5).

**Table 4:** Results of phenolic acid content in *S. officinalis* fermented extract determined using HPLC

Phenolic acid	Concentration mg L^−1^
Chlorogenic acid	347 ± 7
4-Hydroxybenzoic acid	231 ± 8
2,5-Dihydroxybenzoic acid	210 ± 10
Gallic acid	205 ± 6
3-Hydroxybenzoic acid	84 ± 6
Ferulic acid	73 ± 6
3,4-Dihydroxybenzoic acid	36.1 ± 2.1
Vanillic acid	4.9 ± 0.6

**Table 5:** Results of phenolic acid content in acceptor fluid collected during the FSE skin permeation study^a^

Time [h]	Concentration µg cm^−2^ of skin							
	ChA	4-HB	2,5-DHB	GA	3-HB	FA	3,4-DHB	VA
1–4	n.d.	n.d.	n.d.	n.d.	n.d.	n.d.	n.d.	n.d.
5	n.d.	n.d.	n.d.	11.62 ± 1.05	n.d.	n.d.	n.d.	n.d.
6	n.d.	n.d.	0.99 ± 0.21	14.5 ± 1.1	n.d.	n.d.	n.d.	n.d.
8	n.d.	n.d.	2.4 ± 0.7	16.7 ± 1.1	n.d.	n.d.	3.9 ± 0.4	n.d.
24	n.d.	23.3 ± 2.1	5.22 ± 0.22	35.8 ± 1.6	n.d.	n.d.	6.3 ± 0.8	0.25 ± 0.05

a ChA – chlorogenic acid; 4-HB – 4-hydroxybenzoic acid; 2,5-DHB – 2,5-dihydroxybenzoic acid; GA – gallic acid; FA – ferulic acid; 3,4-DHB – 3,4-dihydroxybenzoic acid; VA – vanillic acid; n.d. – not detected.

In FSE, chlorogenic acid, 4-hydroxybenzoic acid, 2,5-dihydroxybenzoic acid, and gallic acid were present in the highest concentrations. Furthermore, permeation studies indicated that gallic acid penetrated the skin at the highest concentration and fastest rate among all identified acids, as it was already present in the acceptor fluid collected in the fifth hour of the analysis. Similar analyses were performed for the ethanol extract of the herb *S. officinalis*. During the preliminary analyses by Muzykiewicz-Szymańska *et al.*,^6^ the phenolic acid content was assessed for an extract obtained by ultrasound-assisted extraction for 60 minutes using a 70% aqueous ethanol solution and a dry extract concentration of 500 g L^−1^. In the ethanol extract, the highest concentrations of gallic acid (424 ± 10 mg L^−1^), vanillic acid (270 ± 5 mg L^−1^), 3,4-dihydroxybenzoic acid (222 ± 9 mg L^−1^), 2,3-dihydroxybenzoic acid (207 ± 7 mg L^−1^), and 2,5-dihydroxybenzoic acid (169 ± 15 mg L^−1^) were identified. Additionally, small amounts of caffeic acid (21 ± 3 mg L^−1^) and chlorogenic acid (13 ± 3 mg L^−1^) were identified.^6^ Comparing these results to the fermented extract of the great burnet herb, we can observe a lower content of gallic acid, 3,4-dihydroxybenzoic acid, 2,5-dihydroxybenzoic acid, and vanillic acid in the FSE, as well as the absence of caffeic and 2,3-dihydroxybenzoic acids identified in the ethanol extract. However, we observe a much higher content of chlorogenic acid (over 25 times higher) and the presence of unidentified 4-hydroxybenzoic acid (231 ± 8 mg L^−1^), 3-hydroxybenzoic acid (84 ± 6 mg L^−1^), and ferulic acid (73 ± 6 mg L^−1^) in the ethanol extract. Additionally, compared to the ethanol extract, in which no penetration of gallic acid through the skin was recorded, from the obtained FSE this acid penetrated the fastest and at the highest concentration – 11.62 ± 1.05 µg cm^−2^ in the 5th hour of permeation to 35.8 ± 1.6 µg cm^−2^ after 24 hours of permeation, which finally constitutes a percentage permeation value of 2.18%. Additionally, in the case of the fermented extract, a higher concentration of 3,4-dihydroxybenzoic acid and 2,5-dihydroxybenzoic acid was identified in the acceptor fluid, the percentage of which was 1.74% and 0.25%, respectively. The presence of 4-hydroxybenzoic acid was also noted, whose concentration in the acceptor fluid after 24 hours of permeation was 23.3 ± 2.1 µg cm^−2^ (percentage permeation equals 1%). It should also be noted that 3,4-dihydroxybenzoic acid and 2,5-dihydroxybenzoic acid were already present in the acceptor fluid at the 8th and 6th hours of permeation, respectively. The presented results confirm the authors' conclusions that the fermentation process changes the profile of phenolic compounds.^17,57^ Fermentation changes the profile of biologically active compounds by converting macronutrients and releasing antioxidant peptides and phenolic compounds. The influence of the bacterial culture used has a major impact on which phenolic compounds will be transformed and how. LAB metabolise phenolic acids through strain-specific decarboxylase and reductase activity. The fermentation process using most *L. plantarum* strains can reduce the concentration of protocatechuic acid (3,4-dihydroxybenzoic acid) by almost 70%.^61^ We made a similar observation by comparing the content of 3,4-hydroxybenzoic acid in FSE and in the ethanol extract analysed in previous studies.^6^ Selected strains of *L. plantarum* and *L. spicheri* also cause degradation of caffeic acid, which was also noticed in our own research. Caffeic acid, which was present in the ethanol extract,^6^ was not identified in the fermented extract. Cinnamic acid and *p*-coumaric acid can be metabolised to 4-hydroxybenzoic acid,^62^ which may be the reason why 4-hydroxybenzoic acid, unidentified in the ethanol extract of great burnet herb, was present in FSE (231 ± 8 mg L^−1^). Yang *et al.*^40^ mention that with increasing fermentation time, hydroxycinnamates are hydrolysed by esterase to hydroxycinnamic acid–caffeic acid, *p*-coumaric acid, and ferulic acid. This phenomenon may explain the presence of ferulic acid in FSE, which was not present in the ethanol extract. Compared to the ethanol extract, FSE contained more than half the amount of gallic acid and a significantly higher (over 25-fold) amount of chlorogenic acid.^6^ Yang *et al.*^63^ observed that fermentation with various lactic acid bacteria generally increases the content of chlorogenic acid in honeysuckle liquid. The authors explain this phenomenon by the chemical decomposition of anthocyanins. Hegde *et al.*,^64^ in their study on the fermentation of cereal bran using *Aspergillus niger*, noted complete degradation of gallic and protocatechuic acid in the experimental sample after 96 hours of the process. The authors suggest that these phenolic acids are degraded by the induction of various phenolic acid esterases. This phenomenon was likely related to the oxidation of these acids or their utilization by fermenting microbes for various purposes. Hunaefi *et al.*^65^ emphasise the ability of *L. plantarum* to degrade many types of phenolic acids, including gallic and protocatechuic acids. Their metabolism involves decarboxylation and reduction of phenolic acids. The decreases in gallic acid and 3,4-dihydroxybenzoic acid content observed in our study may therefore be a consequence of these types of transformations.

The effect of changing the phenolic compound profile is most often a change in the antioxidant activity of the fermented extract. Most often, authors observe an increase in antioxidant potential and total polyphenol content in fermented extracts compared to non-fermented extracts.^17,66^ Michalak-Tomczyk *et al.*^17^ emphasise that this tendency can be explained by the production of metabolites and the increased release of phenolic compounds from the matrix due to the degradation of the original compounds through fermentation. However, these parameters depend on the nature of the plant material and the unique capabilities of the specific starter culture used. Comparing the antioxidant activity of the obtained FSE with the optimised ethanol extract of the *S. officinalis* herb, which was obtained in previous studies by Muzykiewicz-Szymańska *et al.*,^7^ it can be seen that the antioxidant activity of the fermented extract assessed by the FRAP method is more than twice as high as that of the ethanol extract (44.1 ± 0.1 and 20.8 ± 0.9 mmol FeSO_4_ per L, respectively). A higher total polyphenol content was also observed (3.2 ± 0.1 and 2.4 ± 0.1 g GA per L, respectively, for the fermented and ethanol extracts obtained by ultrasound-assisted extraction). A different trend was noted in the case of antioxidant activity assessed by the DPPH method – the ethanol extract showed higher activity (12.7 ± 0.9 mmol Trolox per L), while FSE showed an antioxidant potential of 8.1 ± 0.3 mmol Trolox per L. However, Michalak-Tomczyk *et al.*^17^ point out the differences between methods for assessing antioxidant activity. As an example, they discuss the DPPH and ABTS methods. While the ABTS method accounts for both lipophilic and hydrophilic compounds, the DPPH technique is particularly sensitive to hydrophilic substances. An additional advantage of the fermented extract is the presence of lactic acid, which, in addition to the already mentioned beneficial effect on the skin,^24^ can act as a promoter of the penetration of active compounds through the skin.^67^ This effect may be evidenced by faster skin penetration of selected phenolic acids contained in the fermented extract of the *S. officinalis* herb, compared to the ethanol extract.^6^ The discrepancies in optimal parameters depending on the plant material and bacterial strain used confirm the validity of optimising the fermentation parameters for a specific plant material. Using inappropriate parameters can significantly reduce the efficiency and effectiveness of the process, as well as reduce the biological activity of the obtained extracts, including antioxidant activity and lactic acid content. However, the presented study has certain limitations, including the evaluation of antioxidant activity using selected *in vitro* techniques (DPPH and FRAP), while omitting other important activities, such as the ability to reduce copper ions or scavenge the ABTS radical. Another limitation of the study may be the inability to compare other fermentation techniques for *S. officinalis* due to the lack of available literature data. Further *in vitro*, *ex vivo*, and *in vivo* analyses of the cosmetic and pharmacological potential of the obtained fermented extract are also necessary.

## Conclusions

Optimising *S. officinalis* fermentation parameters for antioxidant activity, total polyphenol content, and lactic acid was successfully conducted using the response surface method. The results obtained for the adopted assumptions indicate a clear range of optimal parameters—time, plant material content, inoculation level, and molasses content—and their impact on the potential of the tested fermented extract. The research we present is the first and preliminary analysis of the great burnet herb fermentation process, confirming its high antioxidant potential and polyphenol content. The HPLC analysis of the content of selected phenolic acids, as well as the *ex vivo* analysis of the permeation of these compounds through porcine skin, initially confirms the dermocosmetic potential of the obtained fermented extract. Due to the potential use of the obtained fermented extract as an active ingredient in topical skin preparations, both cosmetic and pharmaceutical, further research is necessary, including detailed determination of its phytochemical profile. Investigation of its anti-ageing potential, anti-inflammatory and antimicrobial effects, as well as confirmation of its safety, is also planned.

## Author contributions

Conceptualization, Anna Muzykiewicz-Szymańska (A. M.-S.); methodology, A. M.-S., Edyta Kucharska (E. K.), Robert Pełech (R. P.), Anna Nowak (A. N.) and Łukasz Kucharski (Ł. K.); validation, A. M.-S., E. K., and R. P.; formal analysis, A. M.-S., E. K., A. N. and R. P.; investigation, A. M.-S., E. K., and Ł. K.; resources A. M.-S.; data curation, A. M.-S.; writing—original draft preparation, A. M.-S. and E. K.; writing—review and editing, A. M.-S. and E. K.; visualization, A. M.-S., and E. K.; supervision, A. M.-S., and R. P.; project administration, A. M.-S. All authors have read and agreed to the published version of the manuscript.

## Conflicts of interest

There are no conflicts to declare.

## Supplementary Material

RA-015-D5RA06662J-s001

## Data Availability

The data supporting this article have been included as part of the supplementary information (SI). Supplementary information is available. See DOI: https://doi.org/10.1039/d5ra06662j.
