# Supplementary material for: Application of response surface methodology for obtaining fermented extract of Sanguisorba officinalis L. herb with high antioxidant activity, polyphenols, and lactic acid content: analysis of the content and skin permeation of selected phenolic acids
Source: RSC Adv. 2025 Nov 17;15(52):44597–610. doi: 10.1039/d5ra06662j (PMC12622214; doi:10.1039/d5ra06662j)
Supplement: RA-015-D5RA06662J-s001 [file RA-015-D5RA06662J-s001.pdf]

Supplementary Information (SI) for RSC Advances.
This journal is © The Royal Society of Chemistry 2025

Supplementary Information

Application of Response Surface Methodology for obtaining fermented extract of *Sanguisorba officinalis* L. herb with high antioxidant activity, polyphenols, and lactic acid content: analysis of the content and skin permeation of selected phenolic acids.

Anna Muzykiewicz-Szymańska, Edyta Kucharska, Łukasz Kucharski, Robert Pełech, and Anna Nowak

Table S1. Experimental plan and results of the fermentation process of the *S. officinalis* herb, considering the coded and actual values of independent variables.

**X_1** t [-]	**X_2** Inoc [-]	**X_3** MC [-]	**X_4** PMC [-]	**X_1** t [day]	**X_2** Inoc [%]	**X_3** MC [%]	**X_4** PMC [g/L]	**Y_1** AA-FRAP [mmol $FeSO_4$/L]	**Y_2** TPC [g GA/L]	**Y_3** AA-DPPH [mmol Trolox/L]	**Y_4** LAc [g/L]
-1	-1	-1	-1	2	5	5	0.1	1.5	2	1	7
-1	-1	-1	0	2	5	5	1.05	3.0	14	5	19
-1	-1	-1	1	2	5	5	2	5.5	18	6	21
-1	-1	0	-1	2	5	12.5	0.1	0.8	5	2	12
-1	-1	0	0	2	5	12.5	1.05	6.9	20	6	22
-1	-1	0	1	2	5	12.5	2	11.8	24	6	23
-1	-1	1	-1	2	5	20	0.1	1.2	7	3	15
-1	-1	1	0	2	5	20	1.05	10.3	23	6	23
-1	-1	1	1	2	5	20	2	16.5	26	7	23
-1	0	-1	-1	2	15	5	0.1	0.3	6	3	13
-1	0	-1	0	2	15	5	1.05	3.0	21	6	22
-1	0	-1	1	2	15	5	2	5.5	25	6	23
-1	0	0	-1	2	15	12.5	0.1	0.8	11	4	18
-1	0	0	0	2	15	12.5	1.05	6.9	26	7	23
-1	0	0	1	2	15	12.5	2	11.8	28	7	24
-1	0	1	-1	2	15	20	0.1	1.2	15	5	20
-1	0	1	0	2	15	20	1.05	10.3	27	7	24
-1	0	1	1	2	15	20	2	16.5	29	7	24
-1	1	-1	-1	2	25	5	0.1	0.3	9	4	16
-1	1	-1	0	2	25	5	1.05	3.0	24	6	23
-1	1	-1	1	2	25	5	2	5.5	27	7	23
-1	1	0	-1	2	25	12.5	0.1	0.8	15	5	20
-1	1	0	0	2	25	12.5	1.05	6.9	27	7	24
-1	1	0	1	2	25	12.5	2	11.8	29	7	24
-1	1	1	-1	2	25	20	0.1	1.2	18	6	21
-1	1	1	0	2	25	20	1.05	10.3	28	7	24
-1	1	1	1	2	25	20	2	16.5	29	7	24
0	-1	-1	-1	8	5	5	0.1	1.2	7	3	15
0	-1	-1	0	8	5	5	1.05	10.3	23	6	23
0	-1	-1	1	8	5	5	2	16.5	26	7	23
0	-1	0	-1	8	5	12.5	0.1	2.9	13	5	19
0	-1	0	0	8	5	12.5	1.05	19.7	27	7	23
0	-1	0	1	8	5	12.5	2	27.8	28	7	24
0	-1	1	-1	8	5	20	0.1	4.5	17	5	21
0	-1	1	0	8	5	20	1.05	25.6	28	7	24
0	-1	1	1	8	5	20	2	33.5	29	7	24
0	0	-1	-1	8	15	5	0.1	1.2	15	5	20
0	0	-1	0	8	15	5	1.05	10.3	27	7	24
0	0	-1	1	8	15	5	2	16.5	29	7	24
0	0	0	-1	8	15	12.5	0.1	2.9	21	6	22
0	0	0	0	8	15	12.5	1.05	19.7	29	7	24
0	0	0	1	8	15	12.5	2	27.8	29	7	24
0	0	1	-1	8	15	20	0.1	4.5	24	6	23
0	0	1	0	8	15	20	1.05	25.6	29	7	24
0	0	1	1	8	15	20	2	33.5	30	7	24
0	1	-1	-1	8	25	5	0.1	1.2	18	6	21
0	1	-1	0	8	25	5	1.05	10.3	28	7	24

0	1	-1	1	8	25	5	2	16.5	29	7	24
0	1	0	-1	8	25	12.5	0.1	2.9	24	6	23
0	1	0	0	8	25	12.5	1.05	19.7	29	7	24
0	1	0	1	8	25	12.5	2	27.8	30	7	24
0	1	1	-1	8	25	20	0.1	4.5	26	7	23
0	1	1	0	8	25	20	1.05	25.6	30	7	24
0	1	1	1	8	25	20	2	33.5	30	7	24
1	-1	-1	-1	14	5	5	0.1	2.1	11	4	18
1	-1	-1	0	14	5	5	1.05	15.6	26	7	23
1	-1	-1	1	14	5	5	2	23.3	28	7	24
1	-1	0	-1	14	5	12.5	0.1	4.8	18	5	21
1	-1	0	0	14	5	12.5	1.05	26.7	28	7	24
1	-1	0	1	14	5	12.5	2	34.5	29	7	24
1	-1	1	-1	14	5	20	0.1	7.3	21	6	22
1	-1	1	0	14	5	20	1.05	32.5	29	7	24
1	-1	1	1	14	5	20	2	39.3	29	7	24
1	0	-1	-1	14	15	5	0.1	2.1	19	6	21
1	0	-1	0	14	15	5	1.05	15.6	28	7	24
1	0	-1	1	14	15	5	2	23.3	29	7	24
1	0	0	-1	14	15	12.5	0.1	4.8	24	6	23
1	0	0	0	14	15	12.5	1.05	26.7	29	7	24
1	0	0	1	14	15	12.5	2	34.5	30	7	24
1	0	1	-1	14	15	20	0.1	7.3	26	7	23
1	0	1	0	14	15	20	1.05	32.5	30	7	24
1	0	1	1	14	15	20	2	39.3	30	7	24
1	1	-1	-1	14	25	5	0.1	2.1	22	6	22
1	1	-1	0	14	25	5	1.05	15.6	29	7	24
1	1	-1	1	14	25	5	2	23.3	29	7	24
1	1	0	-1	14	25	12.5	0.1	4.8	26	7	23
1	1	0	0	14	25	12.5	1.05	26.7	30	7	24
1	1	0	1	14	25	12.5	2	34.5	30	7	24
1	1	1	-1	14	25	20	0.1	7.3	28	7	24
1	1	1	0	14	25	20	1.05	32.5	30	7	24
1	1	1	1	14	25	20	2	39.3	30	7	24

t – fermentation time; Inoc – inoculum content; MC – molasses content; PMC – plant material content

Table S2. Coefficients of regression equations (a_0–a_{14}), values of correlation coefficients (R^2), and values of adjusted correlation coefficients ($AdjR^2$) for the responses: AA-FRAP, TPC, AA-DPPH, and LAc.

	**AA-FRAP**	**TPC**	**AA-DPPH**	**LAc**
a_0	-6.90018	-17.0582	-1.61352	1.79886
a_1	0.96303	2.1896	0.43544	1.17729
a_2	-0.06346	-0.0517	-0.00935	-0.02405
a_3	-0.02222	1.0659	0.21124	0.57357
a_4	0.00022	-0.0131	-0.00235	-0.00608
a_5	0.53234	1.2105	0.23995	0.6519
a_6	-0.02283	-0.0175	-0.00316	-0.00816
a_7	8.17749	24.3443	4.55795	11.59253
a_8	-4.13519	-4.8522	-0.81425	-1.94291
a_9	0.00055	-0.014	-0.00348	-0.01079
a_{10}	0.03597	-0.0161	-0.00395	-0.01216
a_{11}	0.75686	-0.3704	-0.08701	-0.24241
a_{12}	0.00044	-0.0081	-0.00192	-0.00581
a_{13}	0.00345	-0.1776	-0.04155	-0.11683
a_{14}	0.40984	-0.201	-0.04689	-0.13204
**R^2**	**0.977**	**0.973**	**0.948**	**0.908**
**$AdjR^2$**	**0.972**	**0.967**	**0.937**	**0.888**

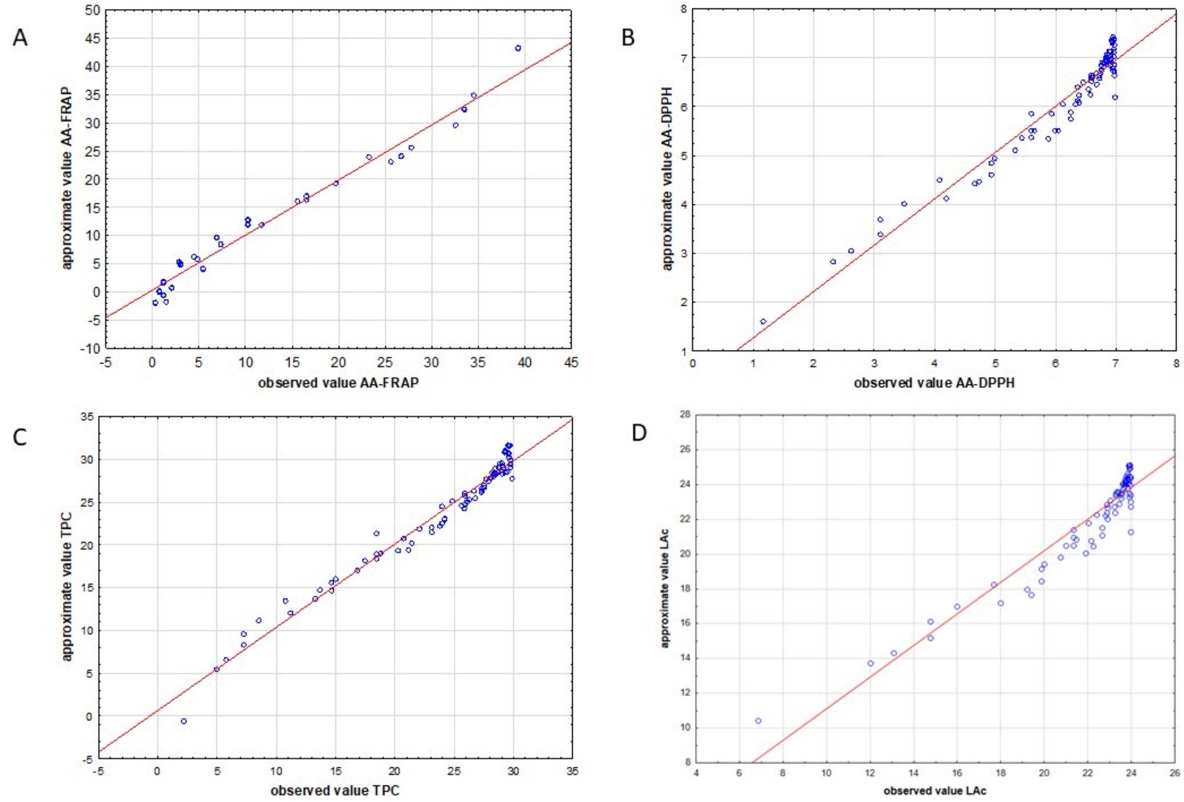

Figure S1. Distribution of the scatter between the approximate and observed values of antioxidant activity determined by the FRAP method (AA-FRAP) (Figure A), the DPPH method (AA-DPPH) (Figure B), total polyphenol content (TPC) (Figure C) and lactic acid content (LAc) (Figure D)

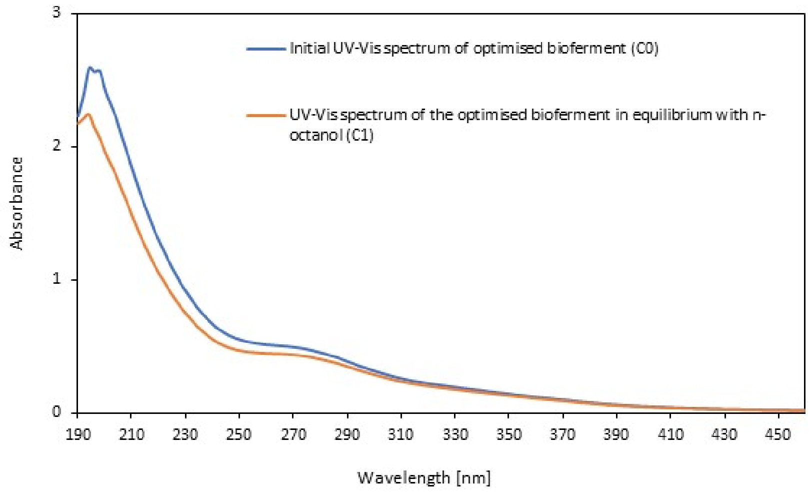

Figure S2. UV-Vis spectrum of the optimised fermented extract from the *S. officinalis* herb
